# Impact of Age-Related Mitochondrial Dysfunction and Exercise on Intestinal Microbiota Composition

**DOI:** 10.1093/gerona/glx197

**Published:** 2017-10-16

**Authors:** David Houghton, Christopher J Stewart, Craig Stamp, Andrew Nelson, Nadim J Aj ami, Joseph F Petrosino, Anil Wipat, Michael I Trenell, Douglass M Turnbull, Laura C Greaves

**Affiliations:** 1 Institute of Cellular Medicine, Newcastle University, Newcastle upon Tyne, UK; 2 Department of Molecular Virology and Microbiology, Alkek Center for Metagenomics and Microbiome Research, Baylor College of Medicine, Houston, Texas; 3 Wellcome Trust Centre for Mitochondrial Research, Institute for Cell and Molecular Biosciences and Institute of Neuroscience, Medical School, Newcastle University, Newcastle upon Tyne; 4 Faculty of Health and Life Sciences, Northumbria University, Newcastle upon Tyne; 5 School of Computing Science, Newcastle University, Newcastle upon Tyne; 6 LLHW Newcastle University Centre for Ageing and Vitality, Faculty of Medicine, Newcastle upon Tyne, UK

**Keywords:** Mitochondria, COX deficiency, Exercise, Gut microbiota

## Abstract

Mitochondrial dysfunction is prevalent in the aging gastrointestinal tract. We investigated whether mitochondrial function in aging colonic crypts and exercise influences microbial gut communities in mice. Twelve *PolgA*^*mut*^/^*mut*^ mice were randomly divided into a sedentary and exercise group at 4 months. Seven-aged matched *PolgA*^*+/+*^ mice remained sedentary throughout. Stool samples were collected at 4, 7, and 11 months, and bacterial profiling was achieved through 16S rRNA sequencing profiling. Mitochondrial enzyme activity was assessed in colonic epithelial crypts at 11 months for *PolgA*^*mut*^/^*mut*^ and *PolgA*^*+/+*^ mice. Sedentary and exercised *PolgA*^*mut*^/^*mut*^ mice had significantly higher levels of mitochondrial dysfunction than *PolgA*^*+/+*^ mice (78%, 77%, and 1% of crypts, respectively). Bacterial profiles of sedentary *PolgA*^*mut*^/^*mut*^ mice were significantly different from the sedentary *PolgA*^*+/+*^ mice, with increases in *Lactobacillus* and *Mycoplasma,* and decreases in *Alistipes, Odoribacter, Anaeroplasma, Rikenella, Parabacteroides,* and *Allobaculum* in the *PolgA*^*mut*^/^*mut*^ mice. Exercise did not have any impact upon gut mitochondrial dysfunction; however, exercise did increase gut microbiota diversity and significantly increased bacterial *genera Mucispirillum* and *Desulfovibrio*. Mitochondrial dysfunction is associated with changes in the gut microbiota. Endurance exercise moderated some of these changes, establishing that environmental factors can influence gut microbiota, despite mitochondrial dysfunction.

Aging is a process characterized by a decline in physiological function, in which mitochondrial DNA (mtDNA) mutations play a key role (1). These mtDNA mutations result in the accumulation of cytochrome *c* oxidase (COX) deficient cells in aging tissues, including the gastrointestinal tract (GIT) (2,3). The colonic epithelium cells are particularly susceptible to age-related mtDNA mutations, in humans on average 15% (40% in extreme cases) having lost normal oxidative phosphorylation (OXPHOS) activity by the age of 70 years (3). The functional consequences of a loss in mitochondrial function in the aging intestinal epithelium are unknown.

The complex nature of the GIT means that any reduction in its function with aging makes it more susceptible to GIT disorders and potentially pathogenic microorganisms (4), which are prevalent in the aging population (5) and primary mitochondrial disorders (6–8). From birth to adulthood, the bacterial community within the GIT, termed “gut microbiota,” adapts with the host working in a symbiotic nature (9).Recent developments in DNA sequencing and bioinformatics (10) have enhanced our understanding of the gut microbiota, and how it contributes toward nutrition, metabolism, immune response, intestinal architecture, and GIT health (11). Therefore, any alteration in the symbiotic relationship between the gut microbiota and host could have potentially harmful effects.

A common GIT disorder is the slower transit time of digesta, which can have a direct impact upon digestion, absorption of nutrients, fermentative processes, defecation, and bacterial excretion (12). This culminates in reduced diversity and an imbalance of potentially healthy and pathogenic bacteria, termed “gut microbiota dysbiosis” (13). For example, in aging and antibiotic-treated patients, bacteria such as *Allobaculum* are reduced, crucial for short chain fatty acids (SCFA) fermentation and its anti-inflammatory and anticarcinogenic properties (14,15). In contrast, *Mucispirillum* and *Desulfovibrio* have been shown to be increased in aging and contribute GIT inflammation and colitis (16). Therefore, the gut microbiota dysbiosis observed in aging (5) can have a profound impact upon human health and quality of life.

Although aging cannot be avoided, manipulation of the gut microbiota to ensure a nondysbiotic state offers an attractive therapeutic approach toward healthy aging and a reduction in GIT disorders. Dietary manipulation has been shown to alter gut microbiota composition (17), improve gut motility (18), and modulate gut barrier function (19). However, far less is known about the effects of physical activity/exercise on the colonic epithelial cells and gut microbiota.

Here, the primary aim of this study was to investigate whether changes in mitochondrial function observed in aging colonic epithelium influence the composition of the gut microbiota. The secondary aim was to investigate whether exercise was able to modulate the gut microbiota composition.

## Materials and Methods

### Murine Model of Aging

To investigate the effect of mitochondrial dysfunction on the gut microbiota composition, we utilized a mouse model (*n* = 19). Twelve mice with a knock-in missense mutation (D257A) at the second endonuclease-proofreading domain of the catalytic subunit of the mtDNA polymerase Polγ (*PolgA*^*mut*^/^*mut*^ mice) were used as an accelerated model of aging, as previously described (20). Mice were age-matched to seven wild-type *PolgA*^*+/+*^ mice which were one generation removed from the experimental mice to avoid the maternal transmission of mtDNA mutations onto a wild-type *PolgA* background (21). All mice were specific pathogen–free and housed in individually ventilated cages based on their retrospective groups (sedentary wild-type, sedentary *PolgA*^*mut*^/^*mu*^, and exercised *PolgA*^*mut*^/^*mut*^) to ensure that there was no cage effect on gut microbiota. Mice were provided with food (RM3 expanded chow) and water ab libitum. Mice were kept in a room with a constant temperature of 25°C with a 12-hour light/dark cycle. All animal experiments were conducted in compliance with the UK Home Office and the Newcastle University Animal Welfare Ethical Review Board.

### Exercise Intervention

At 4 months of age, male *PolgA*^*mut/mut*^ mice were randomly assigned to either the sedentary or endurance exercise groups (exercise [*n* = 7] and sedentary [*n* = 5]), with no mice having previously been subjected to a structured exercise regime. The *PolgA*^*+/+*^ mice were not exercised. The mice were acclimitized to the treadmill for a 1-week period prior to the exercise regime. The speed and duration of exercise was built up over a 2-month period from 17 cm/s for 10 minutes to 20 cm/s for 40 minutes which was continued four times per week until the mice were 11 months of age. A 5-minute warm up and cool down period at 12 cm/s was included. A similar protocol has previously been shown to be well-tolerated and beneficial in the *PolgA*^*mut/mut*^ mice (22). Stool samples were collected at 4, 7, and 11 months to extract DNA and amplify the 16S rRNA gene V4 region. At 11 months of age, the mice were killed by cervical neck dislocation. Collected tissues were removed and flushed with phosphate buffer saline (pH 7.4) to remove any faeces and pellets. Colons were gut longitudinally, placed on a paraffin wax dish, and splayed open and pins inserted around the edges. Tissue was then fixed with 10% neutral buffered formalin for 24 hours. Colons were then rolled into a coil before being transferred to 70% ethanol to await tissue processing and paraffin embedding.

### COX/Succinate Dehydrogenase Histochemistry

Colon samples were collected from all mice at 11 months and then mounted and frozen in isopentane previously cooled to −160°C in liquid nitrogen. Cryostat tissue sections (10 μm) were cut on to glass slides and were then air dried at room temperature for 1 hour. Dual color histochemistry was performed to quantify the proportion of COX deficient crypts present. Sections were incubated in COX medium (100 μM cytochrome C, 4 mM diaminobenzidinetetrahydrochloride, and 20 μg mL−1 catalase in 0.2 M phosphate buffer [PBS] pH 7.0) at 30°C for 50 minutes. Sections were washed in phosphate buffered saline (PBS; 3 × 5 minutes) and then incubated in succinate dehydrogenase (SDH) medium (130 mM sodium succinate, 200 μM phenazinemethosulphate, 1 mM sodium azide, and 1.5 mM nitrobluetetrazolium in 0.2 M PBS pH 7.0) at 37°C for 35 minutes. Colon sections were then washed in PBS (3 × 5 minutes) and dehydrated through graded ethanol (70%, 95%, and 2 × 100%) and two concentrations of Histoclear (National Diagnostics, Atlanta, USA) and mounted in DPX. Slides were scanned using a Scanscope GL scanner (Aperio) and analysed using SpectrumTM software (Aperio), and the percentage of COX deficient colonic crypts were identified in transverse colon sections. Multiple different levels and a minimum of 500 crypts were examined per tissue sample, as previously described (23).

### Bacterial DNA Extraction and 16S rRNA Bacterial Profiling

DNA was extracted from 1 pellet of stool using the Powerlyzer Powersoil DNA isolation kit (MoBio) per the manufactures’ protocol. The 16S rDNA V4 region was amplified by PCR and sequenced in the MiSeq platform (Illumina) using the 2 × 250 bp paired-end protocol yielding pair-end reads that overlap almost completely. The primers used for amplification contain adapters for MiSeq sequencing and single-end barcodes allowing pooling and direct sequencing of PCR products (24).

The 16S rRNA gene pipeline data incorporate phylogenetic and alignment-based approaches to maximize data resolution. The read pairs were demultiplexed based on the unique molecular barcodes, and reads were merged using USEARCH v7.0.1090 (25), allowing zero mismatches and a minimum overlap of 50 bases. Merged reads were trimmed at first base with Q5. In addition, a quality filter was applied to the resulting merged reads and reads containing above 0.05 expected errors were discarded. 16S rRNA gene sequences were clustered into Operational Taxonomic Units (OTUs), a term used to classify groups of bacteria that are closely related at a similarity cutoff value of 97% using the UPARSE algorithm (26). OTUs were mapped to an optimized version of the SILVA Database (27), containing only the 16S V4 region to determine taxonomies. Abundances were recovered by mapping the demultiplexed reads to the UPARSE OTUs. A custom script constructed a rarefied OTU table from the output files generated in the previous two steps for downstream analyses of alpha-diversity, beta-diversity (28), and phylogenetic trends. Raw data are publicly available through the short read archive database under accession number SRP082232.

### Statistical Analysis

Differences in COX/SDH histochemistry between the exercise and sedentary *PolgA*^*mut/mut*^ mice were evaluated using an unpaired *t*-test. Between group differences for weight and Firmicutes:Bacteroidetes ratio were evaluated using an unpaired *t*-test and within group differences using a paired *t*-test (two way). Treatment × Time interactions were assessed using a two-way ANOVA. Statistical significance was set at *p* < .05. Statistical analyses were performed using SPSS statistical analysis software (Version 19, IBM, USA). Analysis and visualization of microbiome communities was conducted in R, utilizing the phyloseq package (29) to import sample data and calculate α- and β-diversity metrics. Each sample was rarefied to 5,000 reads. Significance of categorical variables was determined using the nonparametric Mann–Whitney test for two category comparisons or the Kruskal–Wallis test when comparing three or more categories. Correlation between two continuous variables was determined with linear regression models, where *p*-values indicate the probability that the slope of the regression line is zero. Principal coordinate plots employed the Monte Carlo permutation test (30) to estimate *p*-values. All *p*-values were adjusted for multiple comparisons with the FDR algorithm (31).

## Results

### Polg Mice and Wild-Type Characteristics

**Figure 1.**
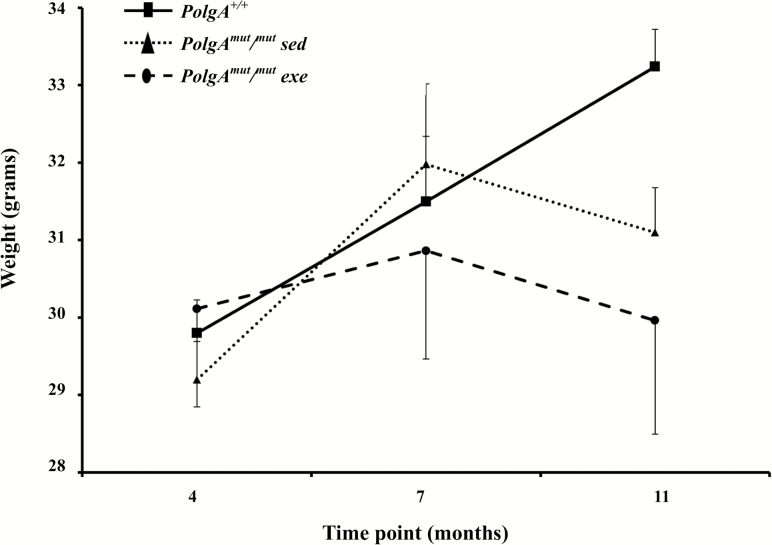
Mean (±*SD*) weights for sedentary *PolgA*^*+/+*^ mice (*n* = 7), sedentary and exercised *PolgA*^mut/mut^ mice (*n* = 7 and 5, respectively) at 4, 7, and 11 months.

A total of 57 samples from months 4, 7, and 11 were included in the 16S rRNA gene based analysis, subdivided into three separate groups: *PolgA*^*mut/mut*^ exercised mice (*n* = 7; mean weight 30 ± 3 g at 4 months), *PolgA*^*mut/mut*^ sedentary mice (*n* = 5; mean weight 29 ± 1 g at 4 months), and sedentary *PolgA*^*+/+*^ mice (*n* = 7; mean weight 30 ± 1 g at 4 months). Both *PolgA*^*mut/mut*^ groups and *PolgA*^*+/+*^ mice were well matched at baseline for weight with no significant differences (*p* > 0.05). Mice in all three groups increased weight between months 4 and 7. However, between months 7 and 11, both *PolgA*^*mut/mut*^ mouse groups began to lose weight (Figure 1), confirming previously published data (20).

### Respiratory Chain Deficiency in Colonic Crypts

**Figure 2.**
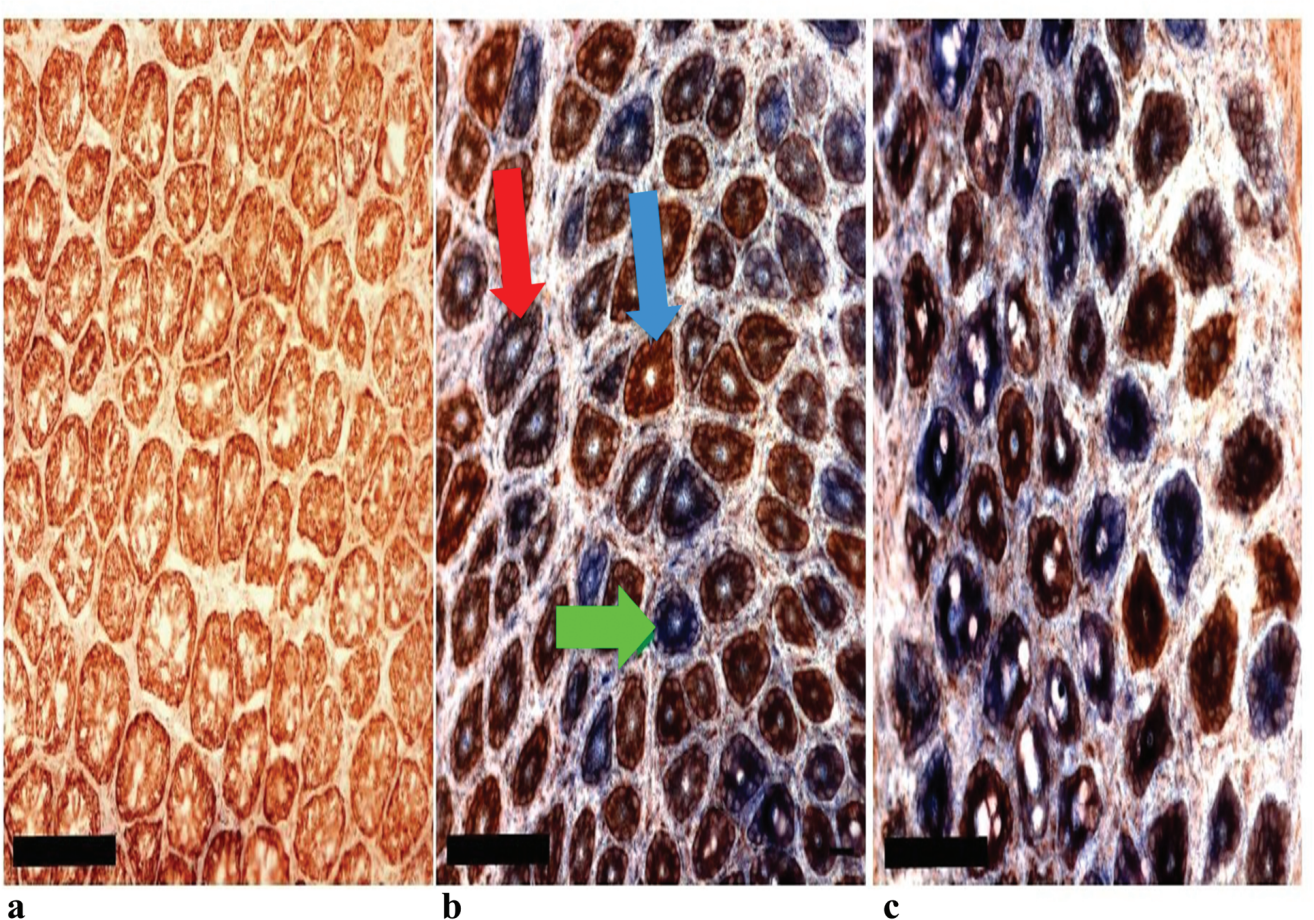
Respiratory chain deficiency in sedentary *PolgA*^*+/+*^ mice (**A**), sedentary *PolgA*^*mut/mut*^ mice (**B**) and exercised *PolgA*^mut/mut^ mice (**C**) colon, showing a transverse section through the crypts. Scale bars: 100 μm. Colonic crypts stained brown are positive for COX activity (blue arrow), those stained blue are deficient for COX activity (green arrow), and crypts stained purple/brown display intermediate COX deficiency (red arrow).

Dual COX/SDH histochemistry was performed on colonic epithelial sections from *PolgA*^*+/+*^ (*n* = 7) and both *PolgA*^*mut/mut*^ mouse groups (sedentary [*n* = 5] and exercised [*n* = 7]) at 11 months. This is a well-established technique to detect respiratory chain deficiency due to mtDNA mutations. COX contains subunits encoded by both the mitochondrial and nuclear genomes, whereas SDH is the catalytic part of complex II and is entirely nuclear encoded. Cells with normal COX enzyme activity are brown, cells which are deficient in COX activity but have normal SDH activity are blue (32). In the *PolgA*^*+/+*^ mice, low levels of COX deficiency were observed (1%). In stark contrast staining in *PolgA*^*mut/mut*^ mice showed both partial and fully COX deficient crypts, which presented as a random mosaic pattern throughout (Figure 2). There was no significant difference in the proportion of COX deficient crypts between the sedentary (mean 78 ± 6%) and exercised (mean 77 ± 13%) *PolgA*^*mut/mut*^ mice (*p* > 0.05).

### Bacterial Profiles

**Figure 3.**
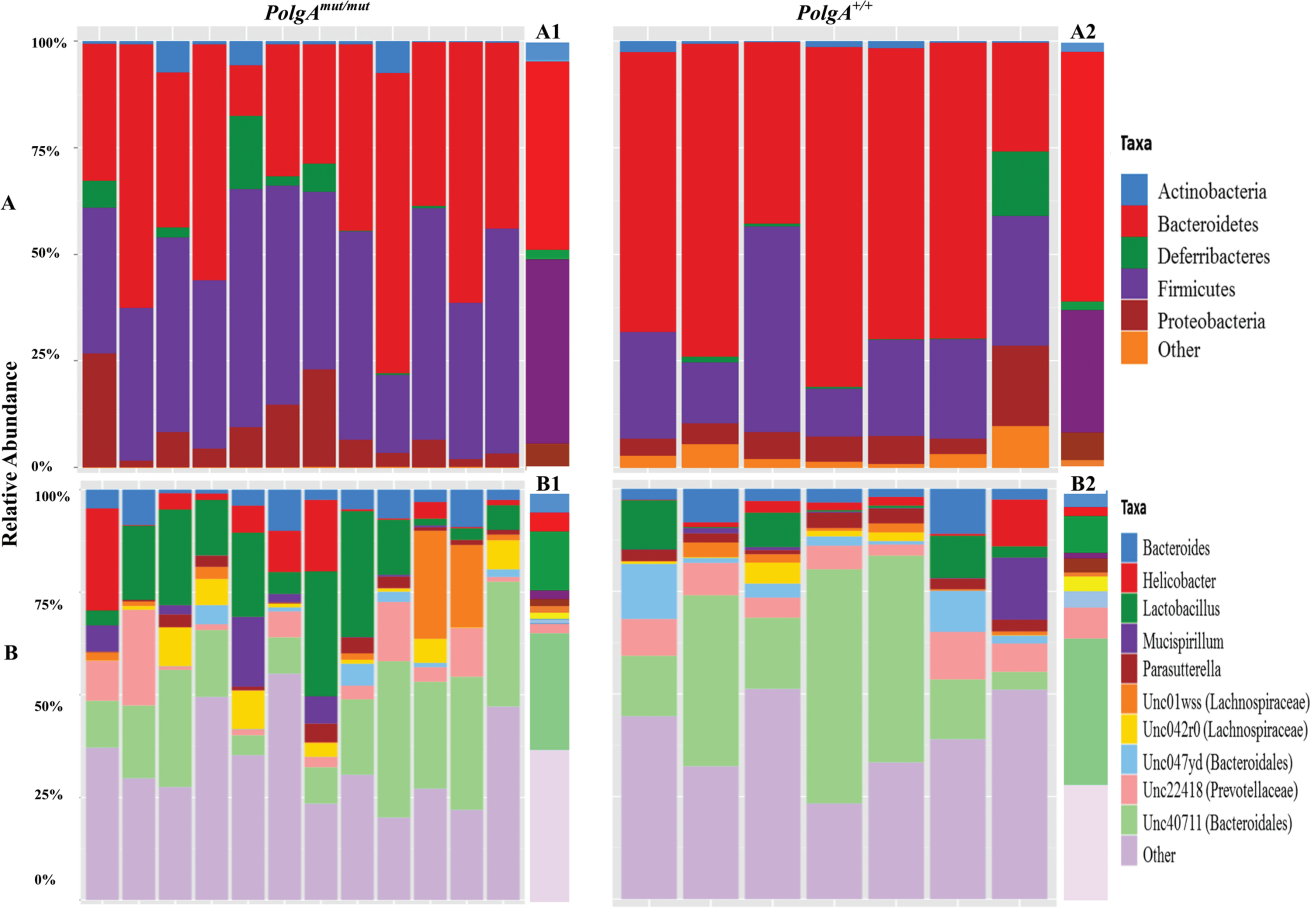
Stacked bar plots showing the distribution of taxa in *PolgA*^mut/mut^ and *PolgA*^*+/+*^ mice at 4 months. *PolgA*^mut/mut^ mice *n* = 12, *PolgA*^*+/+*^ mice *n* =7. Relative abundance at (**A**) phylum level and (**B**) genus level. Group averages at phylum (A1 and A2) and *genus* (B1 and B2) for *PolgA*^mut/mut^ and *PolgA*^*+/+*^ mice, respectively.

**Figure 4.**
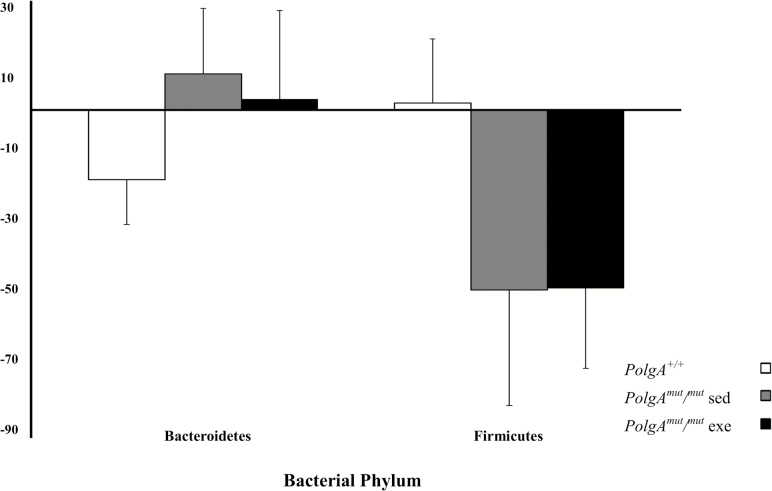
Mean percentage change in Bacteroidetes and Firmicutes between 4 and 11 months for *PolgA*^mut/mut^ sedentary (sed, *n* = 5) and exercise (exe, *n* = 7) and *PolgA*^*+/+*^ mice (*n* = 5) at 4 months.

Bacteroidetes was the most dominant phylum, with the majority of mice having at least 50% relative abundance in both the *PolgA*^*mut/mut*^ and *PolgA*^*+/+*^ groups (Figure 3). Firmicutes was also highly abundant in both groups of mice. At the phylum level, the *PolgA*^*mut/mut*^ mice had lower relative abundance of Bacteroidetes, but higher levels of Firmicutes and Proteobacteria when compared with the *PolgA*^*+/+*^ mice. Over time, the relative abundance of Bacteroidetes increased and Firmicutes reduced in the *PolgA*^*mut/mut*^; however, the opposite was observed in the *PolgA*^*+/+*^ mice, although these changes were nonsignificant (*p* > 0.05; Figure 4). These differences were evident in the Firmicutes/Bacteroidetes ratio of the *PolgA*^*mut/mut*^ mice, which was atypical when compared with the *PolgA*^*+/+*^ mice (Firmicutes/Bacteroidetes ratio; Supplementary Figure 1). There was a significant difference between the *PolgA*^*mut/mut*^ mice and *PolgA*^*+/+*^ at 4 months (*p* = .03), and there were significant within group changes between months 4 and 11 for sedentary and exercise *PolgA*^*mut/mut*^ mice (*p* = .04 and .04, respectively). At the genus level, the *PolgA*^*+/+*^ mice had higher relative abundance of *Bacteroides* and lower levels of *Lactobacillus* and *Helicobacter* (Figure 3).

### Effect of Aging

**Figure 5.**
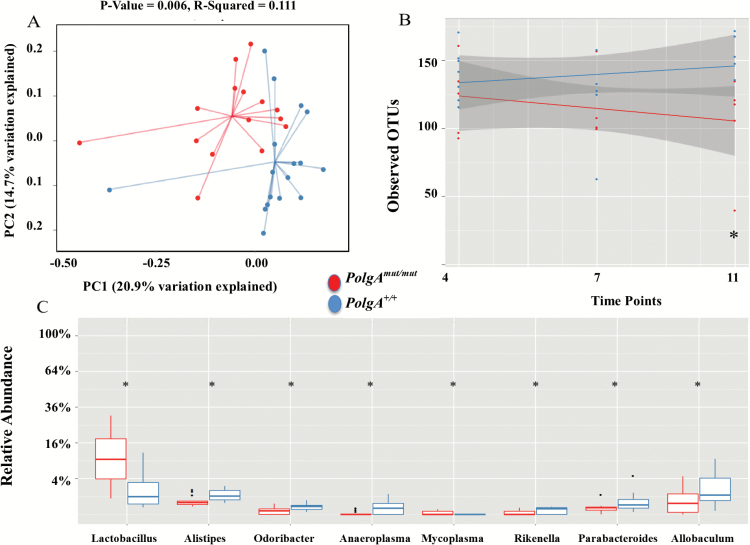
Microbiome for sedentary *PolgA*^mut/mut^ (*n* = 15) and *PolgA*^*+/+*^ mice (*PolgA*^*+/+*^*n* = 21). (**A**) Principle co-ordinate analysis (PCoA) of unweighted UniFrac analysis. (**B**) Regression analysis showing number of observed OTU difference at 4, 7, and 11 months between sedentary *PolgA*^*+/+*^ and *PolgA*^mut/mut^ mice. Grey shading indicates the 95% confidence interval of the mean. (**C**) Boxplots showing the relative abundance of bacteria at genus level at 11 months. * denotes a significant difference between *PolgA*^mut/mut^ and *PolgA*^*+/+*^ mice using Kruskal–Wallis, *p* ≤ .05.

To investigate the effect of the accelerated aging due to mitochondrial dysfunction in the *PolgA*^*mut/mut*^, we compared sedentary *PolgA*^mut/mut^ mice (*n* = 15) vs sedentary *PolgA*^*+/+*^ mice (*n* = 21) bacterial profiles at 4, 7, and 11 months while removing the exercise *PolgA*^mut/mut^ mice. An unweighted UniFrac analysis (accounts for the presence and absence of OTUs and how related the bacteria is by incorporating phylogenetic distances) demonstrated that there was a significant difference (*p* = .006, *R*-squared = 0.111) between *PolgA*^mut/mut^ and *PolgA*^*+/+*^ mice (Figure 5A). Weighted UniFrac (takes into account the number of OTUs and how related the bacteria is by incorporating phylogenetic distances) was not significant (Supplementary Figure 2). α-Diversity was assessed comparing the number of OTUs (a measure of diversity) between *PolgA*^mut/mut^ and *PolgA*^*+/+*^ mice among 4, 7, and 11 months. α-Diversity analysis showed temporal significance (*p* = .01), with an increased number of OTUs in the *PolgA*^*+/+*^ mice compared with the *PolgA*^*mut/mut*^ mice overall, demonstrating that bacterial diversity increased in *PolgA*^*+/+*^ mice, but decreased over time in the *PolgA*^*mut/mut*^ mice (Figure 5B). Although the number of OTUs was similar at 4 months, the number of OTUs gradually decreased between 7 and 11 months in the *PolgA*^*mut/mut*^ mice. The final sample at 11 months revealed a significant reduction (*p* = .007) in the number of OTUs in the *PolgA*^mut/mut^ mice, demonstrating that as the mice age bacterial diversity decreases (Figure 5B). Notably, the Shannon diversity index (a measurement reflecting the number of different bacterial species and how evenly these are distributed in the sample) was not significant within and between *PolgA*^*+/+*^ and *PolgA*^*mut/mut*^ mice at any time point (*p* > 0.05). Comparing the relative abundance between *PolgA*^*+/+*^ and *PolgA*^*mut/mut*^ mice demonstrated significant differences (*p* < .05) between eight taxa at *genus* level (Figure 5C). *Lactobacillus* and *Mycoplasma* were increased and *Alistipes, Odoribacter, Anaeroplasma, Rikenella, Parabacteroides,* and *Allobaculum* were reduced in *PolgA*^*mut/mut*^ mice at 11 months. There were significant changes in *Lactobacillus, Helicobacter,* and *Alistipes* over time between the *PolgA*^*+/+*^ and *PolgA*^*mut/mut*^ mice (*p* > 0.05). Changes in *Lactobacillus, Helicobacter, Alistipes,* and *Allobaculum* over time are presented in Supplementary Figure 3A.

### Effect of Exercise

**Figure 6.**
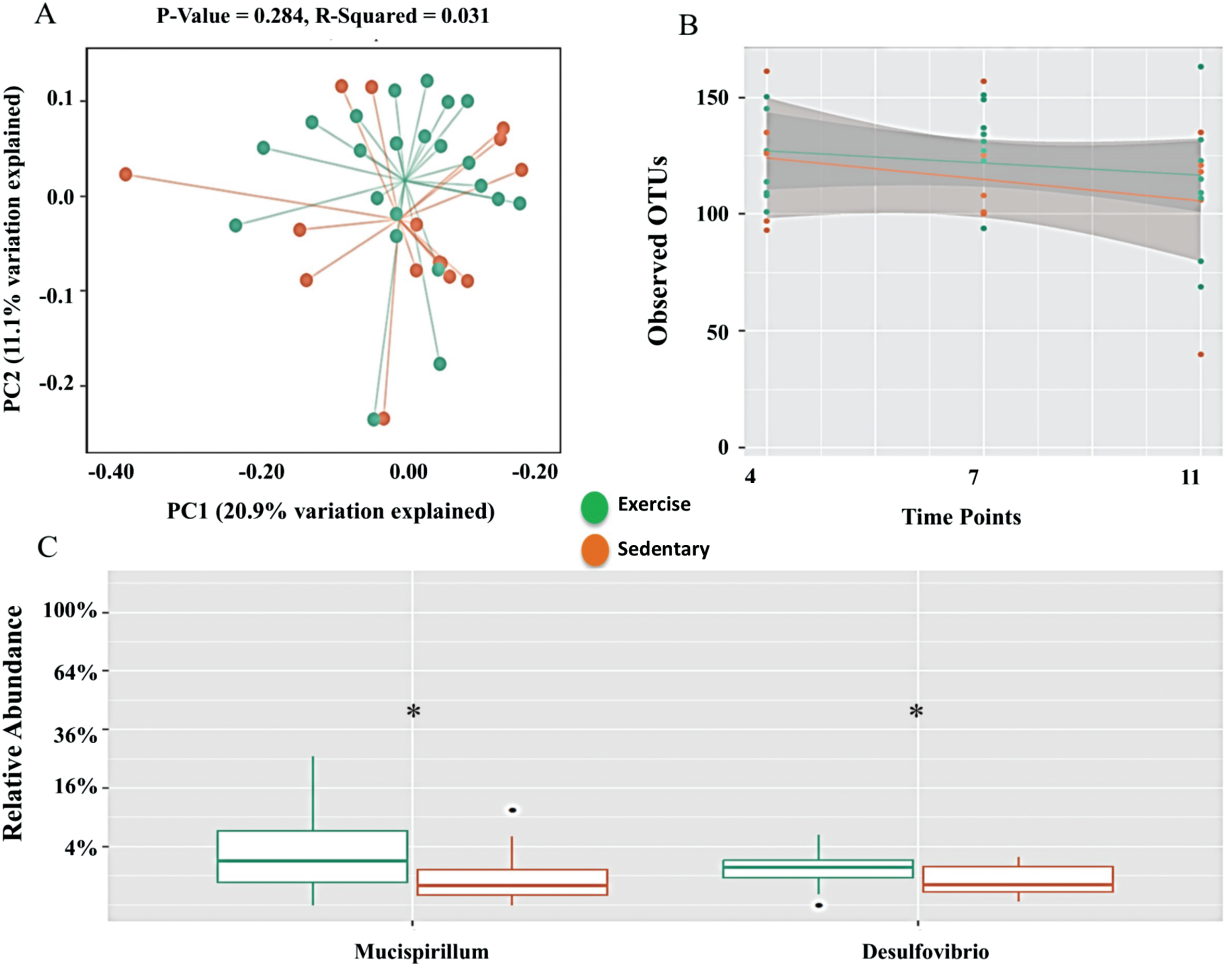
Microbiome for exercise (*n* = 21) and sedentary (*n* = 15) *PolgA*^mut/mut^ mice. (**A**) Principle co-ordinate analysis (PCoA) of unweighted UniFrac analysis. (**B**) Regression analysis showing number of observed OTU difference at 4, 7, and 11 months between sedentary and exercise *PolgA*^mut/mut^ mice. Grey shading indicates the 95% confidence interval of the mean. (**C**) Boxplots showing the relative abundance of bacteria at genus level at 11 months. * represents a significant difference between sedentary and exercise and *PolgA*^mut/mut^ mice using Kruskal–Wallis, *p* ≤ .05.

To evaluate whether exercise was able to modulate the effects of mitochondrial dysfunction on the gut microbiome composition, the sedentary *PolgA*^*mut/mut*^ mice (*n* = 15) were compared with the exercised *PolgA*^*mut/mut*^ mice (*n* = 21). The weighted and unweighted UniFrac analysis demonstrated that there was no significant difference between exercise and sedentary mice (*p* = 0.284, *R*-squared = 0.031 and 0.076, *R*-squared = 0.059, respectively; Figure 6A, weighted UniFrac Supplementary Figure 4A); however, the Bray Curtis dissimilarity (accounts for the abundance of OTUs) was significant (*p* = .02, *R*-squared = 0.073, Supplementary Figure 4B). The α-diversity showed a trend to attenuate the reduction in number of OTUs observed in sedentary *PolgA*^*mut/mut*^ mice; however, this was not significant at 4, 7, and 11 months (*p* > 0.05; Figure 6B), suggesting that exercise may be able to attenuate decreased bacterial diversity observed in aging. There were significant increases in the relative abundance of *Mucispirillum* and *Desulfovibrio* (*p* > 0.05) at month 11 in exercise compared with sedentary *PolgA*^*mut/mut*^ mice (Figure 6C). Within exercise and sedentary *PolgA*^*mut/mut*^ mice, there were changes in the temporal distribution of *Bacteroides*, *Helicobacter*, and *Lactobacillus* from 4 to 11 months (Supplementary Figure 5). There was a significant increase in the relative abundance of *Bacteroides* (*p* = .03), and reductions in both *Helicobacter* and *Lactobacillius* over time in the exercise group, although the latter two were not significant (*p* > 0.05, Supplementary Figure 5). Exercise elicited significant changes in *Mucispirillum* and *Desulfovibrio* over time (*p* > 0.05; Supplementary Figure 3B).

## Discussion

In this study, we have confirmed that *PolgA*^*mut/mut*^ mice display evidence of age-related mitochondrial dysfunction in colonic epithelial crypts, making them a robust model of accelerated aging (20,33). This is the first study to show that the gut microbiota is significantly altered in *PolgA*^*mut/mut*^ mice compared with *PolgA*^*+/+.*^mice, thus demonstrating that age-related mitochondrial dysfunction is able to modulate the gut microbiota. This was manifested as a reduced number of OTUs and diversity. Two of the major taxonomic differences observed here were a significant increase in *Lactobacillus* and a decrease in the relative abundance of *Allobaculum* in the *PolgA*^*mut/mut*^ mice, which has previously been observed in elderly and antibiotic-treated patients (34,35). Intriguingly, the end products of *Allobaculum* fermentation are SCFAs, specifically butyrate which is the primary energy source for colonocytes that promotes epithelial cell health (14). In addition, butyrate has been shown to possess anti-inflammatory and anti-carcinogenic properties (44). Whether mitochondrial dysfunction directly causes the reductions in *Allobaculum is* unclear; however, reductions in a primary energy source such as butyrate through reductions in *Allobaculum* may be contributing to reduced colonic epithelia crypt cell proliferation and increased apoptosis previously reported in *PolgA*^*mut/mut*^ mice (36).

In addition, in the *PolgA*^*mut/mut*^ mouse small intestine, mtDNA mutations were associated with reduced lipid absorption and altered stem progenitor cell cycling (36). The gut microbial composition has been shown to be crucial for macronutrient digestion and absorption; for example, *Bacteroides thetaiotaomicron* has been shown to augment lipid hydrolysis up regulating expression of colipase, which is essential for pancreatic lipase activity (37). Although all mice were provided with the same food and drink ad libitum, the *PolgA*^*mut/mut*^ had a reduction in weight, common in the aging phenotype. Although not recorded, this may have been due to consuming different amounts of food and drink. However, there is evidence demonstrating that exercise increases appetite (38) and muscles mass regardless of aging (39), which would attenuate weight loss. Although it cannot be definitively proven in the current study, this is a preliminary study to identify that mice who present with an aging phenotype have reduced lipid absorption, altered stem progenitor cell cycling (36), due to mitochondrial dysfunction in colonic crypts and alterations in bacterial composition. Reduced lipid absorption and altered stem progenitor cell cycling would undoubtedly contribute to weight loss observed in *PolgA*^*mut/mu*^ mice and have previously been reported in humans (20,33,36). The direction of causality between weight loss and changes in bacterial composition remains unknown, and prospective and longitudinal studies are warranted.

The host immune system is mediated by gut epithelium, commensal bacteria, and immune cells (40), all of which are impaired with aging and associated with poor health (41). Mitochondrial dysfunction is present from early in life and increases with age (42). This may have impact upon the ability of colonic crypts to function efficiently. Defects in the cells of the colonic crypts (enterocytes, goblet, enteroendocrine, and tuft cells) can affect homeostasis of the GIT, impacting upon barrier permeability, nutrient uptake, immune system, and the dynamic cross talk with the gut microbiota (43–45). In this mouse model, it has been shown that mtDNA dysfunction is the primary driving force responsible for the aging phenotype (20,33). However, the resulting gut microbiota dysbiosis may then accentuate the aging phenotype further, as it has been identified as a major risk factor for GIT disorders (43). There was no difference in the level of COX deficiency in the colonic epithelial cells of the exercise and sedentary *PolgA*^*mut/mut*^ mice. Exercise elicits mitochondrial biogenesis in skeletal muscle (46); however, in the present study, exercise had no effect on mitochondrial function in the colonic epithelial cells of the *PolGA*^*mut/mut*^. These data suggest that the age associated decline in physiological function of the GIT may be unavoidable, but modulation of the gut microbiota through lifestyle interventions, including dietary modification, which has been shown to alter bacterial composition within days (17), exercise presented here, and tailored medication may prove effective to maintain GIT homeostasis and ensure healthy aging.

Moderate exercise has been shown to improve immune function and reduce inflammation (15), commonly reported in GIT disorders. Exercise has also been shown to modulate gut microbiota composition and improve GIT health (47,48). However, the effects of exercise on colonic crypts and gut microbiota in an accelerated model of aging have not been observed. In the current study, exercise had no effect on mitochondrial function in the colonic epithelial cells of the *PolgA*^*mut/mut*^ mice. Exercise was able to attenuate the observed reduction in bacterial diversity associated with aging, as measured through an increase in the number of OTUs over time. However, it is unclear as to whether this increase in diversity is entirely beneficial, as previously suggested (47). For example, a decrease in *Helicobacter* and a significant increase in *Bacteroides* suggest a potentially protective effect of exercise. In contrast, exercise elicited significant increases in the relative abundance of *Mucispirillum* and *Desulfovibrio* at month 11. These bacteria have been linked with inflammation, are known mucin degraders, producers of hydrogen sulfide and ammonia (49,50), and have been observed in models of colitis (16). During exercise, blood flow to the GIT is reduced and re-directed to the working muscles. Although not measured here, this may reduce movement of digesta through the GIT, increasing exposure of the GIT to potentially pathogenic bacteria. Furthermore, intense exercise may induce acute GIT symptoms, inducing gut mucosal ischemia and oxidative stress, disrupting epithelial cells (51), and accentuating inflammation (52). The increases in *Mucispirillum* and *Desulfovibrio* seen here may be linked with an inflammatory response to exercise in the *PolgA*^*mut/mut*^ mice. Further research is required to compare various intensities of exercise and assess inflammatory response following exercise in larger sample sizes in various ages to ascertain a safe and beneficial intensity for aging populations to ensure that they remain active as part of healthy aging phenotype.

## Conclusion

This study is the first to investigate the effects of mitochondrial dysfunction on the gut microbiota composition and whether these effects can be influenced by exercise. These data demonstrate significant changes in the gut microbiota in the presence of high levels of COX deficiency. Exercise did not attenuate the degree of COX deficiency; however, exercise did have an effect on the gut microbiota, although not to the same extent as aging. Further work is warranted to assess the effectiveness of environmental factors on modulating the gut microbiota and improving the aging phenotype.

## Availability of Data

Raw data are publicly available through the short read archive database under accession number SRP082232.

## Supplementary Material

Supplementary data is available at *The Journals of Gerontology, Series A: Biological Sciences and Medical Sciences* online.

## Author Contributions

DH: Study concept and design, study execution, data collection, statistical analysis, manuscript preparation, and approval of final draft submitted. CJS: Study concept and design, statistical analysis, manuscript preparation, and approval of final draft submitted. CS: Study concept and design, study execution, data collection, manuscript preparation, and approval of final draft submitted. AN: Study concept and design, critical revision of the paper and approval of final draft submitted. NJA: Study concept and design, critical revision of the paper and approval of final draft submitted. JFP: Study concept and design, critical revision of the paper and approval of final draft submitted. AW: Study concept and design, critical revision of the paper and approval of the final draft submitted. MIT: Study concept and design, critical revision of the paper and approval of the final draft submitted. DMT: Study concept and design, critical revision of the paper and approval of the final draft submitted. LCG: Study concept and design, study execution, data collection, critical revision of the paper and approval of final draft submitted.

## Funding

The research leading to these results has received funding from the European Union Seventh Framework Programme (FP7/2007–2013) under grant agreement Health-F2-2009–241762, for the project FLIP; Newcastle University Centre for Brain Ageing and Vitality (supported by the Biotechnology and Biological Sciences Research Council, Engineering and Physical Sciences Research Council, Economic and Social Research Council and Medical Research Council [G0700718]), The UK National Institute for Health Research Biomedical Research Centre on Ageing and Age Related Diseases; and Diabetes UK.

## Conflict of Interest

None reported.

## Supplementary Material

Supplementary_Figure_1

Supplementary_Figure_2

Supplementary_Figure_3

Supplementary_Figure_4

Supplementary_Figure_5

## References

[1] Kirkwood TB Understanding the odd science of aging. Cell. 2005;120:437–447. doi: 10.1016/j.cell.2005.01.027. 15734677 10.1016/j.cell.2005.01.027

[2] McDonald SA, Greaves LC, Gutierrez-Gonzalez L et al Mechanisms of field cancerization in the human stomach: the expansion and spread of mutated gastric stem cells. Gastroenterology. 2008;134:500–510. doi: 10.1053/j.gastro.2007.11.035. 18242216 10.1053/j.gastro.2007.11.035

[3] Taylor RW, Barron MJ, Borthwick GM et al Mitochondrial DNA mutations in human colonic crypt stem cells. J Clin Invest. 2003;112:1351–1360. doi: 10.1172/JCI19435. 14597761 10.1172/JCI19435 PMC228466

[4] Saffrey MJ Aging of the mammalian gastrointestinal tract: a complex organ system. Age (Dordr). 2014;36:9603. doi: 10.1007/s11357-013-9603-2. 24352567 10.1007/s11357-013-9603-2 PMC4082571

[5] Claesson MJ, Cusack S, O’Sullivan O et al Composition, variability, and temporal stability of the intestinal microbiota of the elderly. Proc Natl Acad Sci U S A. 2011;108 Suppl 1:4586–4591. doi: 10.1073/pnas.1000097107. 20571116 10.1073/pnas.1000097107 PMC3063589

[6] Biagi E, Nylund L, Candela M et al Through ageing, and beyond: gut microbiota and inflammatory status in seniors and centenarians. PLoS One. 2010;5:e10667. doi: 10.1371/journal.pone.0010667. 20498852 10.1371/journal.pone.0010667 PMC2871786

[7] O’Mahony D, O’Leary P, Quigley EM Aging and intestinal motility: a review of factors that affect intestinal motility in the aged. Drugs Aging. 2002;19:515–527. 12182688 10.2165/00002512-200219070-00005

[8] Giordano C, Sebastiani M, De Giorgio R et al Gastrointestinal dysmotility in mitochondrial neurogastrointestinal encephalomyopathy is caused by mitochondrial DNA depletion. Am J Pathol. 2008;173:1120–1128. doi: 10.2353/ajpath.2008.080252. 18787099 10.2353/ajpath.2008.080252 PMC2543079

[9] Ley RE, Peterson DA, Gordon JI Ecological and evolutionary forces shaping microbial diversity in the human intestine. Cell. 2006;124:837–848. doi: 10.1016/j.cell.2006.02.017. 16497592 10.1016/j.cell.2006.02.017

[10] Round JL, Mazmanian SK The gut microbiota shapes intestinal immune responses during health and disease. Nat Rev Immunol. 2009;9:313–323. doi: 10.1038/nri2515. 19343057 10.1038/nri2515 PMC4095778

[11] Hooper LV, Gordon JI Commensal host-bacterial relationships in the gut. Science. 2001;292:1115–1118. 11352068 10.1126/science.1058709

[12] Macfarlane GT, Cummings JH, Macfarlane S, Gibson GR Influence of retention time on degradation of pancreatic enzymes by human colonic bacteria grown in a 3-stage continuous culture system. J Appl Bacteriol. 1989;67:520–527. 2480341

[13] Claesson MJ, Jeffery IB, Conde S et al Gut microbiota composition correlates with diet and health in the elderly. Nature. 2012;488:178–184. doi: 10.1038/nature11319. 22797518 10.1038/nature11319

[14] Donohoe DR, Garge N, Zhang X et al The microbiome and butyrate regulate energy metabolism and autophagy in the mammalian colon. Cell Metab. 2011;13:517–526. doi: 10.1016/j.cmet.2011.02.018. 21531334 10.1016/j.cmet.2011.02.018 PMC3099420

[15] Walsh NP, Gleeson M, Shephard RJ et al Position statement. Part one: Immune function and exercise. Exerc Immunol Rev. 2011;17:6–63 21446352

[16] Rooks MG, Veiga P, Wardwell-Scott LH et al Gut microbiome composition and function in experimental colitis during active disease and treatment-induced remission. ISME J. 2014;8:1403–1417. doi: 10.1038/ismej.2014.3. 24500617 10.1038/ismej.2014.3 PMC4069400

[17] Walker AW, Ince J, Duncan SH et al Dominant and diet-responsive groups of bacteria within the human colonic microbiota. ISME J. 2011;5:220–230. doi: 10.1038/ismej.2010.118. 20686513 10.1038/ismej.2010.118 PMC3105703

[18] Kashyap PC, Marcobal A, Ursell LK et al Complex interactions among diet, gastrointestinal transit, and gut microbiota in humanized mice. Gastroenterology. 2013;144:967–977. doi: 10.1053/j.gastro.2013.01.047. 23380084 10.1053/j.gastro.2013.01.047 PMC3890323

[19] Cani PD, Possemiers S, Van de Wiele T et al Changes in gut microbiota control inflammation in obese mice through a mechanism involving GLP-2-driven improvement of gut permeability. Gut. 2009;58:1091–1103. doi: 10.1136/gut.2008.165886. 19240062 10.1136/gut.2008.165886 PMC2702831

[20] Trifunovic A, Wredenberg A, Falkenberg M et al Premature ageing in mice expressing defective mitochondrial DNA polymerase. Nature. 2004;429:417–423. doi: 10.1038/nature02517. 15164064 10.1038/nature02517

[21] Ross JM, Stewart JB, Hagström E et al Germline mitochondrial DNA mutations aggravate ageing and can impair brain development. Nature. 2013;501:412–415. doi: 10.1038/nature12474. 23965628 10.1038/nature12474 PMC3820420

[22] Safdar A, Bourgeois JM, Ogborn DI et al Endurance exercise rescues progeroid aging and induces systemic mitochondrial rejuvenation in mtDNA mutator mice. Proc Natl Acad Sci USA. 2011;108:4135–4140. doi: 10.1073/pnas.1019581108. 21368114 10.1073/pnas.1019581108 PMC3053975

[23] Baines HL, Stewart JB, Stamp C et al Similar patterns of clonally expanded somatic mtDNA mutations in the colon of heterozygous mtDNA mutator mice and ageing humans. Mech Ageing Dev. 2014;139:22–30. doi: 10.1016/j.mad.2014.06.003. 24915468 10.1016/j.mad.2014.06.003 PMC4141908

[24] Caporaso JG, Lauber CL, Walters WA et al Ultra-high-throughput microbial community analysis on the Illumina HiSeq and MiSeq platforms. ISME J. 2012;6:1621–1624. doi: 10.1038/ismej.2012.8. 22402401 10.1038/ismej.2012.8 PMC3400413

[25] Edgar RC Search and clustering orders of magnitude faster than BLAST. Bioinformatics. 2010;26:2460–2461. doi: 10.1093/bioinformatics/btq461. 20709691 10.1093/bioinformatics/btq461

[26] Edgar RC UPARSE: highly accurate OTU sequences from microbial amplicon reads. Nat Methods. 2013;10:996–998. doi: 10.1038/nmeth.2604. 23955772 10.1038/nmeth.2604

[27] Quast C, Pruesse E, Yilmaz P et al The SILVA ribosomal RNA gene database project: improved data processing and web-based tools. Nucleic Acids Res. 2013;41(Database issue):D590–D596. doi: 10.1093/nar/gks1219. 23193283 10.1093/nar/gks1219 PMC3531112

[28] Lozupone C, Knight R UniFrac: a new phylogenetic method for comparing microbial communities. Appl Environ Microbiol. 2005;71:8228–8235. doi: 10.1128/AEM.71.12.8228-8235.2005. 16332807 10.1128/AEM.71.12.8228-8235.2005 PMC1317376

[29] McMurdie PJ, Holmes S phyloseq: an R package for reproducible interactive analysis and graphics of microbiome census data. PLoS One. 2013;8:e61217. doi: 10.1371/journal.pone.0061217. 23630581 10.1371/journal.pone.0061217 PMC3632530

[30] Dwass M Modified randomization tests for nonparametric hypotheses. Ann. Math. Stat. 1957;28:181–187.

[31] Benjamini Y, Hochberg Y Controlling the false discovery rate: a practical and powerful approach to multiple testing. J Royal Stat Soc. 1995;57:289–300.

[32] Old SL, Johnson MA Methods of microphotometric assay of succinate dehydrogenase and cytochrome c oxidase activities for use on human skeletal muscle. Histochem J. 1989;21:545–555. 2556354 10.1007/BF01753355

[33] Kujoth GC, Hiona A, Pugh TD et al Mitochondrial DNA mutations, oxidative stress, and apoptosis in mammalian aging. Science. 2005;309:481–484. doi: 10.1126/science.1112125. 16020738 10.1126/science.1112125

[34] O’Sullivan O, Coakley M, Lakshminarayanan B et al; ELDERMET Consortium Alterations in intestinal microbiota of elderly Irish subjects post-antibiotic therapy. J Antimicrob Chemother. 2013;68:214–221. doi: 10.1093/jac/dks348. 22949626 10.1093/jac/dks348

[35] Woodmansey EJ, McMurdo ME, Macfarlane GT, Macfarlane S Comparison of compositions and metabolic activities of fecal microbiotas in young adults and in antibiotic-treated and non-antibiotic-treated elderly subjects. Appl Environ Microbiol. 2004;70:6113–6122. doi: 10.1128/AEM.70.10.6113-6122.2004. 15466557 10.1128/AEM.70.10.6113-6122.2004 PMC522128

[36] Fox RG, Magness S, Kujoth GC, Prolla TA, Maeda N Mitochondrial DNA polymerase editing mutation, PolgD257A, disturbs stem-progenitor cell cycling in the small intestine and restricts excess fat absorption. Am J Physiol Gastrointest Liver Physiol. 2012;302:G914–G924. doi: 10.1152/ajpgi.00402.2011. 22345551 10.1152/ajpgi.00402.2011 PMC3362078

[37] Hooper LV, Wong MH, Thelin A, Hansson L, Falk PG, Gordon JI Molecular analysis of commensal host-microbial relationships in the intestine. Science. 2001;291:881–884. doi: 10.1126/science.291.5505.881. 11157169 10.1126/science.291.5505.881

[38] Pomerleau M, Imbeault P, Parker T, Doucet E Effects of exercise intensity on food intake and appetite in women. Am J Clin Nutr. 2004;80:1230–1236. 15531670 10.1093/ajcn/80.5.1230

[39] Sipilä S, Suominen H Effects of strength and endurance training on thigh and leg muscle mass and composition in elderly women. J Appl Physiol (1985). 1995;78:334–340. 7713834 10.1152/jappl.1995.78.1.334

[40] Sansonetti PJ, Medzhitov R Learning tolerance while fighting ignorance. Cell. 2009;138:416–420. doi: 10.1016/j.cell.2009.07.024. 19665961 10.1016/j.cell.2009.07.024

[41] Biagi E, Candela M, Fairweather-Tait S, Franceschi C, Brigidi P Aging of the human metaorganism: the microbial counterpart. Age (Dordr). 2012;34:247–267. doi: 10.1007/s11357-011-9217-5. 21347607 10.1007/s11357-011-9217-5 PMC3260362

[42] Greaves LC, Nooteboom M, Elson JL et al Clonal expansion of early to mid-life mitochondrial DNA point mutations drives mitochondrial dysfunction during human ageing. PLoS Genet. 2014;10:e1004620. doi: 10.1371/journal.pgen.1004620. 25232829 10.1371/journal.pgen.1004620 PMC4169240

[43] Berger E, Rath E, Yuan D et al Mitochondrial function controls intestinal epithelial stemness and proliferation. Nat Commun. 2016;7:13171. doi: 10.1038/ncomms13171. 27786175 10.1038/ncomms13171 PMC5080445

[44] Man AL, Gicheva N, Nicoletti C The impact of ageing on the intestinal epithelial barrier and immune system. Cell Immunol. 2014;289:112–118. doi: 10.1016/j.cellimm.2014.04.001. 24759078 10.1016/j.cellimm.2014.04.001

[45] Rera M, Clark RI, Walker DW Intestinal barrier dysfunction links metabolic and inflammatory markers of aging to death in Drosophila. Proc Natl Acad Sci USA. 2012;109:21528–21533. doi: 10.1073/pnas.1215849110. 23236133 10.1073/pnas.1215849110 PMC3535647

[46] Wright DC, Han DH, Garcia-Roves PM, Geiger PC, Jones TE, Holloszy JO Exercise-induced mitochondrial biogenesis begins before the increase in muscle PGC-1alpha expression. J Biol Chem. 2007;282:194–199. doi: 10.1074/jbc.M606116200. 17099248 10.1074/jbc.M606116200

[47] Clarke SF, Murphy EF, O’Sullivan O et al Exercise and associated dietary extremes impact on gut microbial diversity. Gut. 2014;63:1913–1920. doi: 10.1136/gutjnl-2013-306541. 25021423 10.1136/gutjnl-2013-306541

[48] Campbell SC, Wisniewski PJ, Noji M et al The effect of diet and exercise on intestinal integrity and microbial diversity in mice. PLoS One. 2016;11:e0150502. doi: 10.1371/journal.pone.0150502. 26954359 10.1371/journal.pone.0150502 PMC4783017

[49] Berry D, Schwab C, Milinovich G et al Phylotype-level 16S rRNA analysis reveals new bacterial indicators of health state in acute murine colitis. ISME J. 2012;6:2091–2106. doi: 10.1038/ismej.2012.39. 22572638 10.1038/ismej.2012.39 PMC3475367

[50] Carbonero F, Benefiel AC, Gaskins HR Contributions of the microbial hydrogen economy to colonic homeostasis. Nat Rev Gastroenterol Hepatol. 2012;9:504–518. doi: 10.1038/nrgastro.2012.85. 22585131 10.1038/nrgastro.2012.85

[51] Zuhl M, Schneider S, Lanphere K, Conn C, Dokladny K, Moseley P Exercise regulation of intestinal tight junction proteins. Br J Sports Med. 2014;48:980–986. doi: 10.1136/bjsports-2012-091585. 23134759 10.1136/bjsports-2012-091585

[52] Schulzke JD, Ploeger S, Amasheh M et al Epithelial tight junctions in intestinal inflammation. Ann N Y Acad Sci. 2009;1165:294–300. doi: 10.1111/j.1749-6632.2009.04062.x. 19538319 10.1111/j.1749-6632.2009.04062.x

