# Supplementary figures and images for: Impact of Age-Related Mitochondrial Dysfunction and Exercise on Intestinal Microbiota Composition
Source: J Gerontol A Biol Sci Med Sci. 2017 Oct 16;73(5):571–8. doi: 10.1093/gerona/glx197 (PMC5905657; doi:10.1093/gerona/glx197)

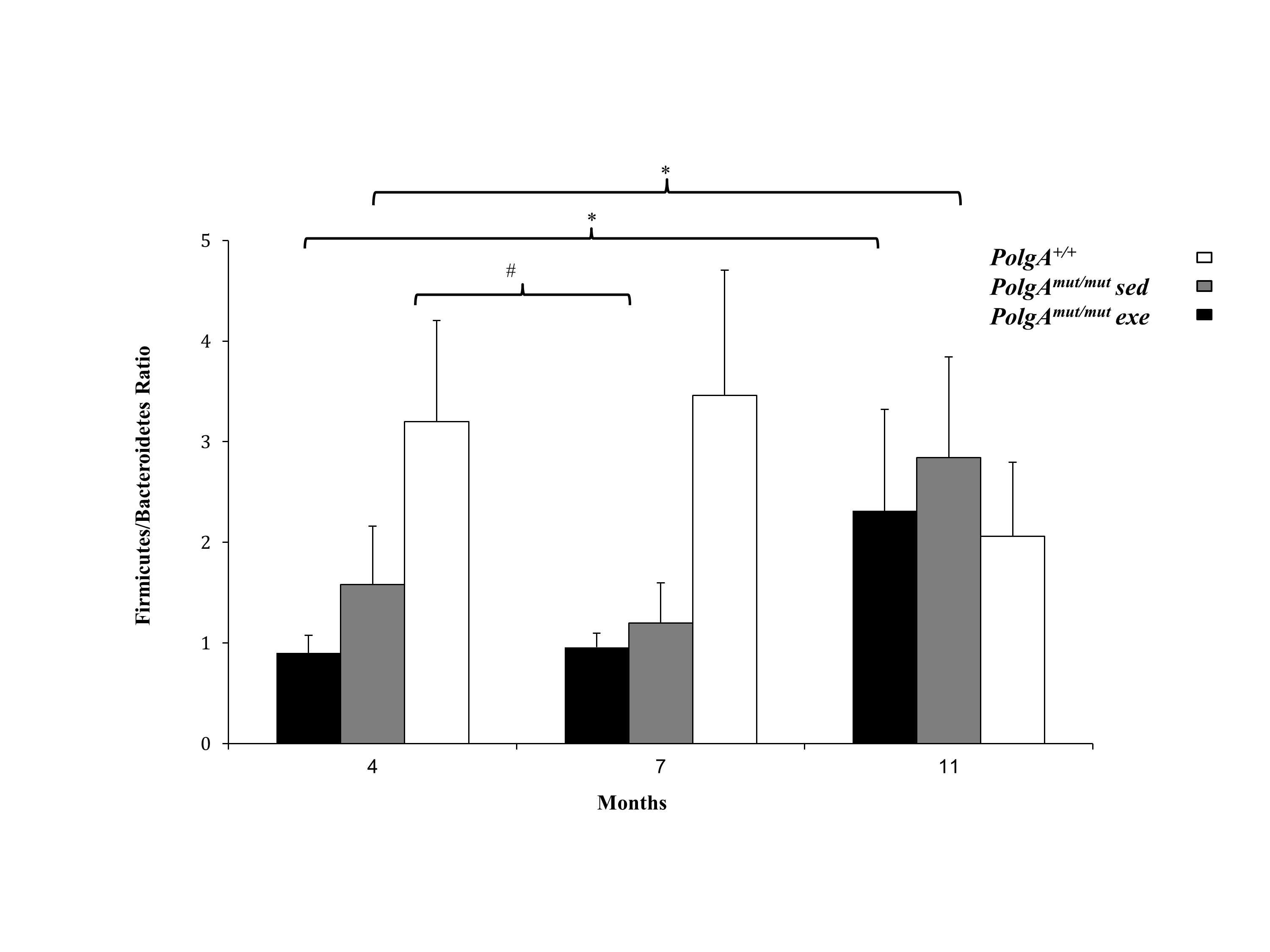

Supplement: Supplementary_Figure_1 [file glx197_suppl_supplementary_figure_1.png]

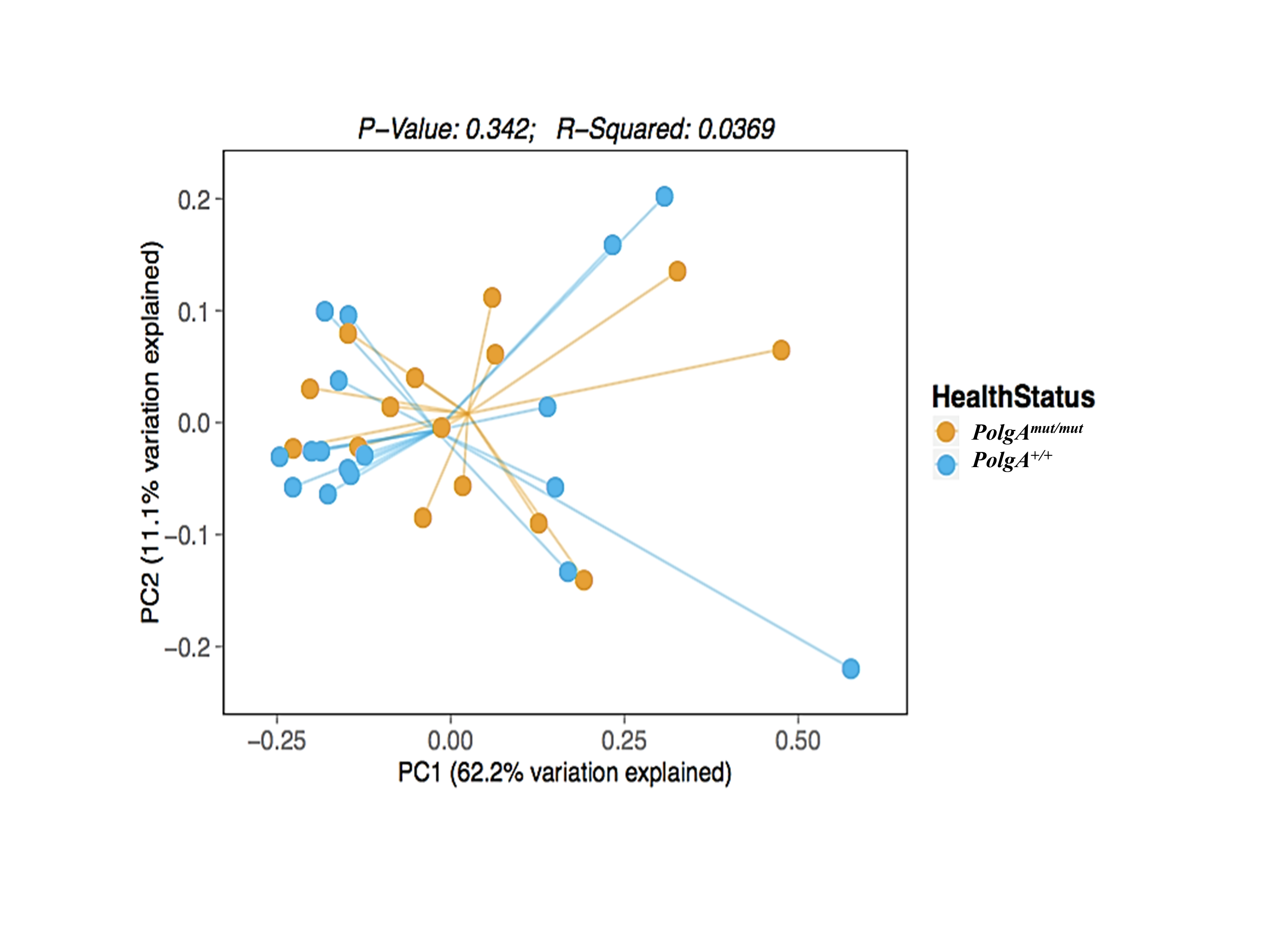

Supplement: Supplementary_Figure_2 [file glx197_suppl_supplementary_figure_2.png]

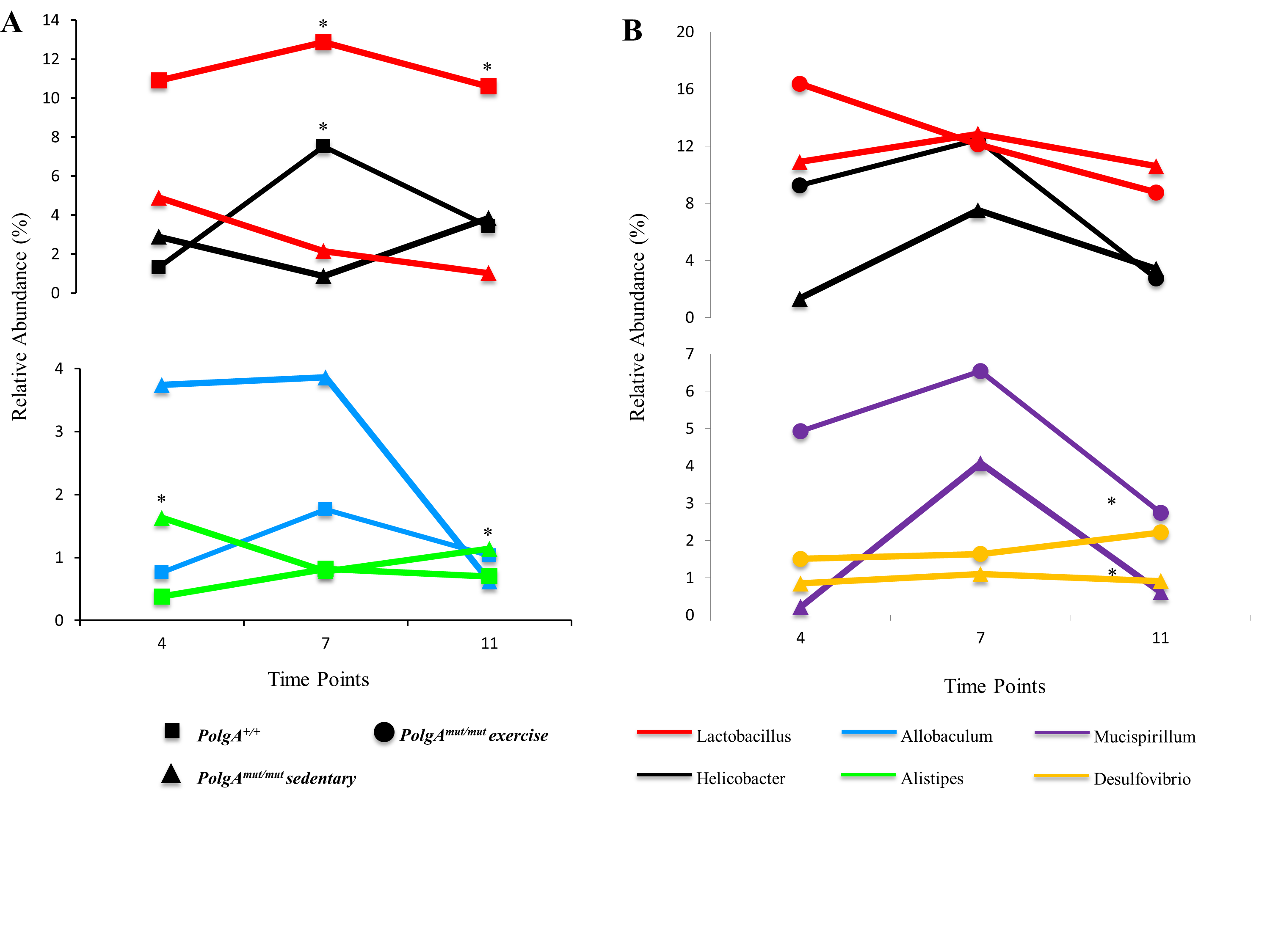

Supplement: Supplementary_Figure_3 [file glx197_suppl_supplementary_figure_3.png]

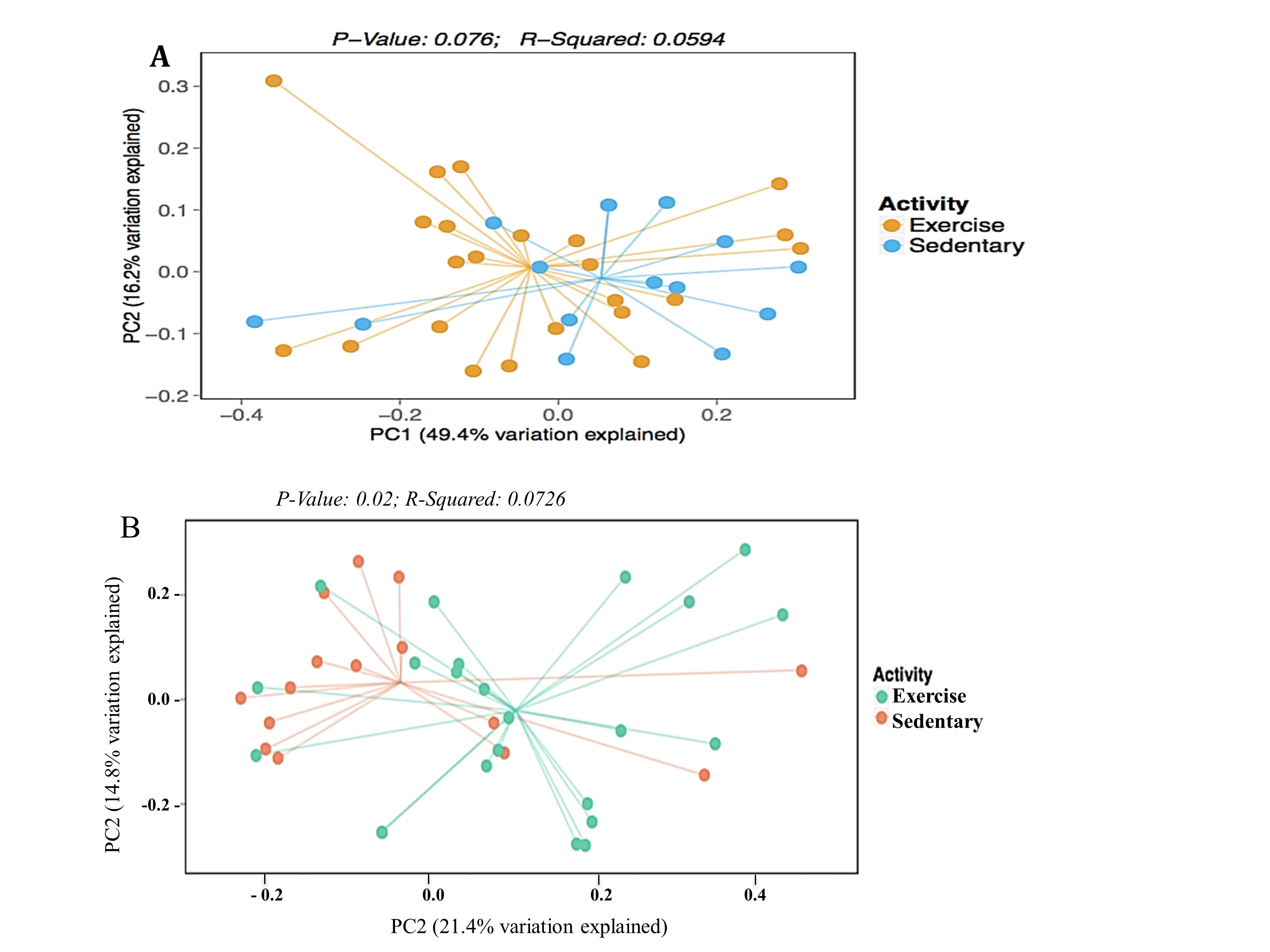

Supplement: Supplementary_Figure_4 [file glx197_suppl_supplementary_figure_4.png]

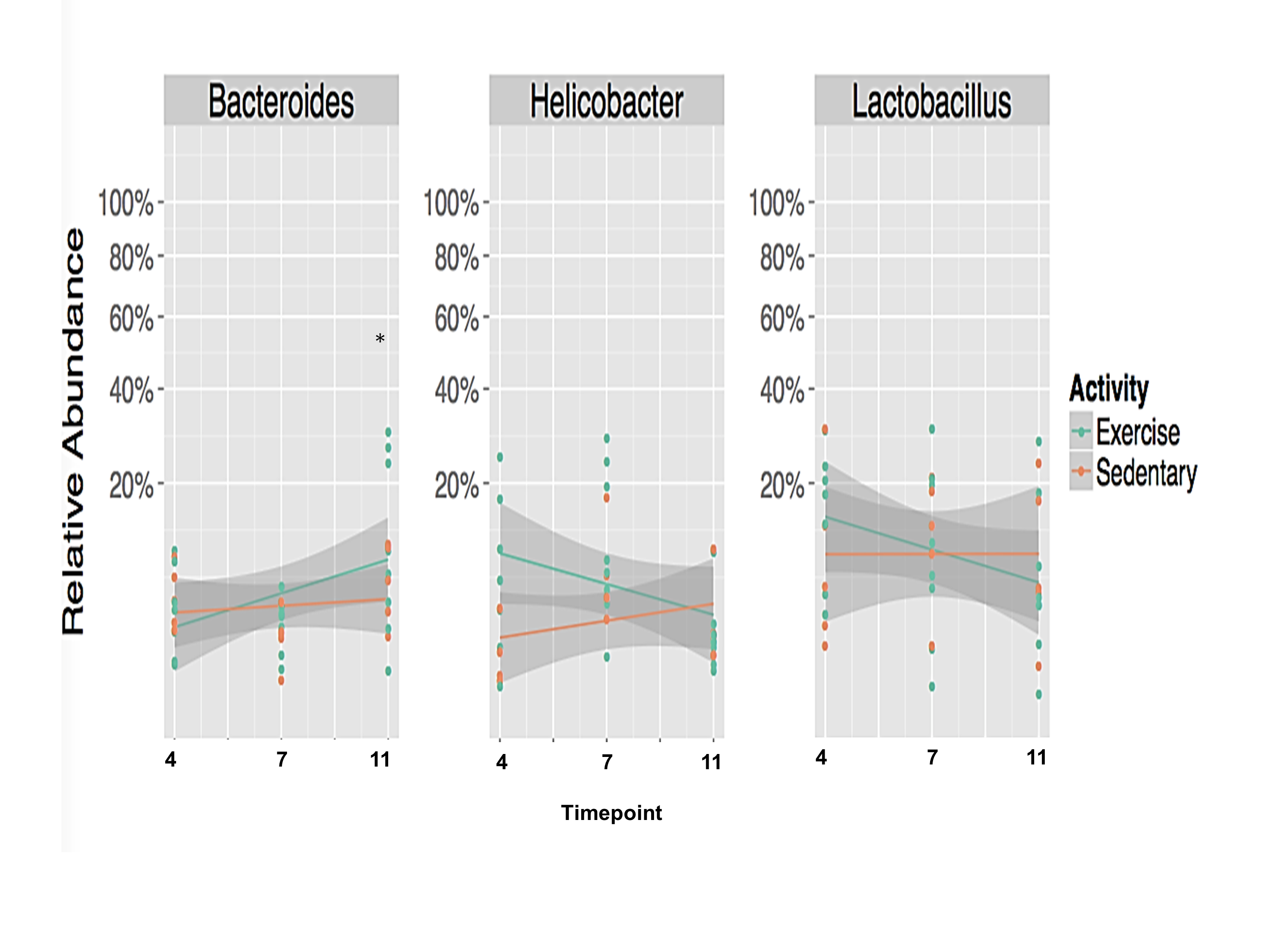

Supplement: Supplementary_Figure_5 [file glx197_suppl_supplementary_figure_5.png]
